# Basolateral activation with TLR agonists induces polarized cytokine release and reduces barrier function in RPE in vitro

**DOI:** 10.1007/s00417-020-04930-2

**Published:** 2020-09-19

**Authors:** Laura Terheyden, Johann Roider, Alexa Klettner

**Affiliations:** University Medical Center, Department of Ophthalmology, University of Kiel, Arnold-Heller-Str. 3, Haus B2, 24105 Kiel, Germany

**Keywords:** Toll-like receptor, RPE, Barrier, IL-1ß, IL-6, TNFα

## Abstract

**Purpose:**

Systemic inflammation may be of importance in the development of AMD. RPE cells can recognize danger signals with toll-like receptors (TLR) and may react in a pro-inflammatory manner. In this study, we evaluated the basal and apical secretions of TNFα, IL-6, and IL-1β in primary RPE cells and RPE/choroid explant cells under basolateral stimulation of TLR2, 3, and 4; the effects on barrier function; and their influence on neuronal cell viability.

**Methods:**

RPE/choroid tissue explants were prepared from porcine eyes and cultivated in modified Ussing chambers; primary porcine RPE cells on transwell plates. Cells were basally stimulated with agonists Pam2CSK4 (Pam; TLR2), polyinosinic/polycytidylic acid (Poly I:C; TLR3), and lipopolysaccharide (LPS; TLR4) for 24 h. Supernatants were evaluated with ELISA for cytokines TNFα, IL-6, and IL-1β. Apical supernatants were applied to SHSY-5Y cells, and cell viability was evaluated in MTT assay. Barrier function was tested by measuring transepithelial electrical resistance (TER) and occludin immunostaining.

**Results:**

None of the tested TLR agonists was toxic on RPE cells after 24 h of exposure. Unstimulated RPE cells secreted hardly any cytokines. Pam induced IL-6, IL-1ß, and TNFα on the basal and apical sides at all concentrations tested. Poly I:C induced IL-6 and TNFα primarily at the basal side at lower but on both sides at higher concentrations. LPS induced IL-6, IL-1ß, and TNFα apically and basally at all concentrations tested. In the RPE/choroid, a strong difference between apical and basal secretions could be found. IL-6 was constitutively secreted basally, but not apically, but was induced by all agonists on both sides. IL-1ß and TNFα alpha were strongly induced on the basal side by all agonists. TER was reduced by all agonists, with Pam and LPS being effective in all concentrations tested. Occludin expression was unaltered, but the distribution was influenced by the agonists, with a less distinct localization at the cell borders after treatment. None of the agonists or supernatants of treated RPE and RPE/choroid organ cultures exerted any effect on viability of SHSY-5Y cells.

**Conclusions:**

Danger signals activating TLRs can induce polarized cytokine expression and contribute to the loss of barrier function in the RPE.

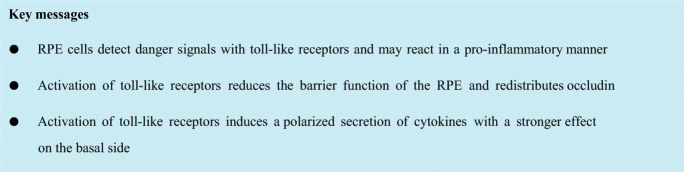



## Introduction

Age-related macular degeneration (AMD) is the most common cause for vision loss in industrialized countries [1]. AMD is a multifactorial disease with inflammation considered to be an important factor in disease progression. The underlying mechanisms, however, are not elucidated so far and still under investigation [2]. The retinal pigment epithelium (RPE) has many tasks in the retina in order to maintain the functionality of the photoreceptors and vision [3]. Among these tasks is the contribution of the RPE to the immune privilege of the eye [4]. RPE cells make up the outer blood–retinal barrier, forming tight junctions, thereby impeding para-cellular transport and cellular infiltration [5]. RPE cells are highly polarized cells, with distinct interactions and function at the respective sides, facing either the neuro-retina or the choroid [6]. These functions include polarized secretion of enzymes or cytokines [7–9]. In addition, the RPE acts as a sentinel for danger signals [4]. It expresses toll-like receptors (TLR), which can detect danger- and pathogen-associated molecular patterns, activating an inflammatory response of the RPE [10–13]. TLRs are discussed to be involved in AMD development. Certain polymorphisms of TLR3 are considered to be associated with AMD, though these findings are under debate and may depend on ethnicity [14–16]. Furthermore, an association was shown between polymorphisms in TLR2 and 4 and AMD development [17, 18]. TLR3 activation may exert toxic effects on RPE cells and increase their VEGF secretion [12, 19]. TLR2 activation in the RPE by *Chlamydia pneumoniae* enhanced choroidal neovascularization [20], while TLR4 may be involved in retinal degeneration and complement mediated RPE cell activation [21, 22]. Furthermore, activation of TLR can induce the secretion of cytokines. The polarization of the secretion or the influence of TLR agonists on barrier function, however, has received little attention so far [11, 23, 24].

AMD is a multifactorial disease, of which systemic inflammation is discussed as a potentially major contributing factor. Studies show elevated systemic cytokine concentrations and inadequate systemic immune modulation [25–29]. In the retina, the RPE is in close contact with the choroid, and because of its sentinel function, it may react to systemic pro-inflammatory signals presented at the choroidal (basal) side, which may have consequences for the inflammatory milieu at the retinal (apical) side.

In order to investigate a potential influence of systemic inflammation on the RPE in vitro, we investigated the effect of a basal (originating from the choroidal side) activation of TLR2, 3, and 4 of the RPE on the secretion of the cytokines IL-6, IL-1ß, and TNFα and on the barrier function of these cells.

## Methods

### Preparation and culture of RPE/choroid explants

**Fig. 1:**
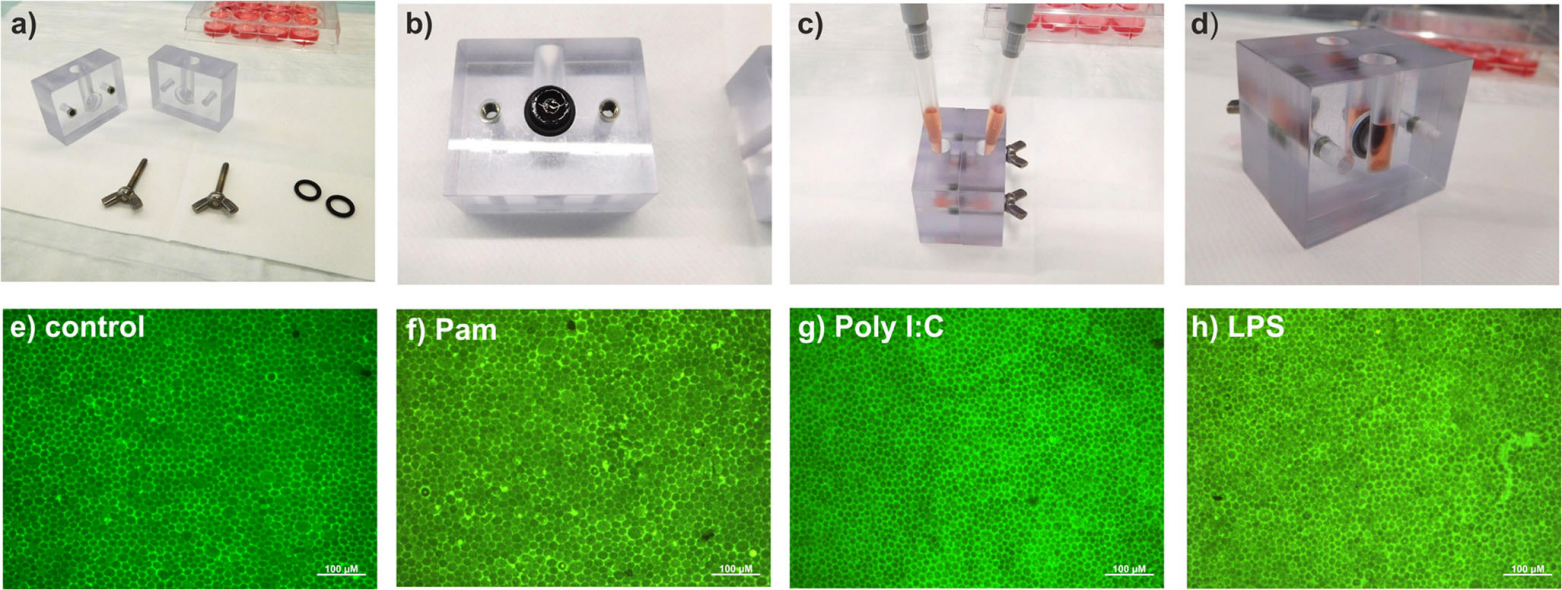
Modified Ussing chamber. **a** Components of the Ussing chamber, including “apical” and “basal” medium reservoir, screws, and sealing rings. **b** RPE/choroid organ culture explant in position on “basal” medium reservoir before assembly. **c** Application of “apical” and “basal” media in assembled chamber. **d** Assembled chamber containing organ culture ring and medium. **e**–**h** representative examples of calcein stains. **e** Untreated control. **f** Treated with 100 ng/ml Pam. **g** Treated with 100 μg/ml Poly I:C. **h** Treated with 10 μg/ml LPS. Scale bar indicates 100 μm

RPE/choroid organ cultures were prepared as previously described [8]. Briefly, porcine eyes from the local abattoir were cleaned of adjacent tissue and immersed in antiseptic solution (Betaisodona, Mundipharma, Limburg, Germany). The anterior segment, vitreous, lens, and neuro-retina were removed, and the RPE/choroid layer was gently separated from the sclera using forceps and scissors. RPE/choroid explants were fixed between the two parts of a fixation ring (modified part no.1300, Minucells and Minutissue, Bad Abbach, Germany), pressed into each other with chalazion pliers. The rings were transferred to 12-well culture plates (Sarstedt AG, Nümbrecht, Germany) on a head plate (37 °C) and allowed to equilibrate in culture medium. The culture medium was a mixture of equal amounts of DMEM and Ham-F12 medium (PAA, Cölbe, Germany) which was supplemented with penicillin/streptomycin (1%), L-glutamine, HEPES (10 mM), sodium pyruvate (1.1%) and porcine serum (10%), taurine (100 μM), and calcium (2 mM) (all PAA). After 24 h of incubation, the cultures were brought into a modified Ussing chamber (build by the Technical Faculty of the University of Kiel). The chamber allows the separation of the apical (RPE-facing) and basal (choroid-facing) compartments (Fig. 1a–d). For quality control, the barrier between the two compartments was verified with Brilliant Blue staining (data not shown). For this, 30 μl of Brilliant Peel (Fluoron GmbH, Ulm, Germany) was added to fresh medium on the basal side of the chamber. After 24 h of cultivation, the staining intensity was photometrically analyzed (*λ* = 584).

### Primary RPE isolation and cell culture

Porcine RPE cells were isolated as previously described [30, 31]. Briefly, porcine eyes were obtained from a local abattoir and prepared within 4 h of death. They were cleaned of adjacent tissue and immersed in antiseptic solution (Betaisodona, Mundipharma, Limburg, Germany). The anterior part of the eye was removed, as well as vitreous, lens, and neuro-retina. In each eye cup, trypsin (0.25% in PBS (Biochrom, Berlin, Germany)) was added and incubated for 10 min at 37 °C. The trypsin solution was removed and substituted with trypsin/EDTA (0.25%/0.02% in PBS) for 45 min at 37 °C. RPE cells were gently pipetted of the choroid, collected in medium, and washed. Cells were seeded on transwell plates (Sarstedt AG, Nümbrecht, Germany; volume of medium: 1 ml apical, 1.5 ml basal) and cultivated in Dulbecco’s modified Eagle’s medium (DMEM, PAA, Cölbe, Germany) supplemented with penicillin/streptomycin (1%), HEPES (25 mM), 1.1% sodium pyruvate, 1% non-essential amino acids (all Biochrom), and 10% fetal calf serum (FCS; LINARISblue, Wertheim-Bettingen, Germany). Cells were used at confluence after primary seeding to avoid dedifferentiation due to passaging [32]. Morphology was assessed in light microscopy. Barrier function was evaluated with the transepithelial electrical resistance (TER) (see below).

### SHSY-5Y cell culture

As a model for neuronal cells, SHSY-5Y cells were used [33]. SHSY-5Y cells were cultivated in flasks with RPMI medium (Merck, Darmstadt, Germany), supplemented with penicillin/streptomycin (1%) and FCS (10%). For their dissemination, cells were washed with PBS and detached from the flasks with biotase (Biochrom). Cells were counted in a Trypan Blue exclusion assay in Neubauer’s cell chamber. For experiments with RPE supernatants, 300,000 cells per well were disseminated on collagen A–coated 24-well plates. For the experiments with organ culture supernatants 10,000 cells per well were seeded on uncoated 96-well plates. When cells reached a confluence of 85%, they were treated for 24 h at 37 °C with supernatants and controls, respectively.

### Transepithelial electrical resistance measurement

The barrier function of the RPE monolayer was detected by measuring the TER in primary porcine RPE cells [34]. The measurement was conducted with an Epithelial Voltohmmeter (EVOM2, World Precision Instruments, Sarasota, FL, USA), using STX2-chopstick electrodes. TER was evaluated using the formula: TER = (measurement − blank value) (Ω) × membrane area (cm^2^).

### Experimental treatment of RPE cells

After 3 weeks of cultivation and a stable TER (140.8 ∓ 55.4 Ω*cm^2^), cell culture medium was renewed, and primary RPE cells were stimulated on the basal side with TLR2 agonist Pam2CSK4 (Pam; 10, 50, or 100 ng/ml), TLR3 agonist polyinosinic/polycytidylic acid (Poly I:C; 1, 10, or 100 μg/ml), or TLR4 agonist lipopolysaccharide (LPS; 0.1, 1, or 10 μg/ml) for 24 h. TER was measured again, and supernatants were collected, centrifuged for 15 min at 4 °C and 13,000 rpm, and stored at − 20 °C until further evaluation. In order to investigate cell viability, an MTT assay was conducted.

### MTT assay

MTT assay is an established viability assay [35] and was conducted as previously described [36]. In brief, after respective treatment, MTT reagent (0.5 mg/ml MTT (3-(4,5-dimethylthiazol-2-yl)-2,5-diphenyltetrazoliumbromid; Sigma-Aldrich, solved in DMEM without phenol red (GE Healthcare, München, Germany)) was applied for 2 hours at 37 °C. DMSO was added to lyse the cells. When measuring SHSY-5Y cells, this protocol was varied [37]. Here, MTT supernatants were collected in Eppendorf reaction tubes and centrifuged. Supernatant was discarded and pellets harvested. Pellets were re-administered to their corresponding wells, in which the cells were then lysed with DMSO. After the application of DMSO, in both approaches, the absorbance at *λ* = 570 nm was measured with a microplate reader (ELx800, BioTek Instruments, Bad Friedrichshall, Germany). Each MTT experiment was independently repeated 3 times.

### Experimental treatment of RPE/choroid organ culture

Twenty-four hours after preparation, TLR agonists Pam (TLR2; 100 ng/ml), Poly I:C (TLR3; 100 μg/ml), or LPS (TLR4; 10 μg/ml) were added for 24 h. Basal and apical supernatants were collected, centrifuged for 15 min at 4 °C and 13,000 rpm, and stored at − 20 °C until further evaluation. To assess the viability of the cultures, at the end of each experiment, a calcein stain was conducted.

### Calcein stain

It is highly recommended to assess the viability of the RPE cells of the RPE/choroid organ cultures before including them into the further evaluation [38]. Therefore, calcein stains were conducted as previously described [39]. In brief, after the experiments, the RPE/choroid organ culture rings were incubated with calcein AM (AnaSpec, Inc., San Jose, CA) for 30 min, washed with Dulbecco’s PBS (PAA), and observed with a fluorescence microscope (*λ*ex/*λ*em = 497/517 nm (Zeiss, Jena, Germany)). Only rings that displayed a viability of more than 90% were included in the evaluation. Representative calcein stains are shown in Fig. 1(e–h).

### Occludin staining

Occludin is a member of RPE tight junction complex [40]. Occludin staining was conducted as previously described with modifications [41]. The tissue was washed with cold PBS and fixed with 4% formaldehyde for 15 min. After washing with PBS three times, cells were permeabilized with 5% Triton-X 100 for 10 min at room temperature. The tissue was blocked with 1% BSA in PBS for 20 min at room temperature and incubated with the primary antibody (rabbit occludin polyclonal antibody, Thermo Fisher Scientific Inc., Rockford, IL, USA, diluted 1:100 in 2.5% Triton-X 100 and 1% BSA containing PBS) at 4 °C overnight. The tissue was washed with PBS three times and incubated for 1 h with a second antibody (Alexa Fluor 555 goat anti-rabbit, Thermo Fisher Scientific Inc., Rockford, IL, USA, diluted 1:1000 in Roti-Immunoblot diluted 1:10 with aqua dest and Hoechst for nucleus staining diluted 1:500). After washing with PBS three times, either the organ culture explant was removed from the ring or the transwell culture membranes were cut out with a scalpel. The tissue was mounted onto a glass side, covered with mounting solution, and observed with a fluorescence microscope (*λ*ex/*λ*em = 554/570). Each experiment was independently repeated 3 times. From each slide, five pictures were taken and evaluated.

### ELISA

The basal and apical supernatants of both organ culture and primary RPE transwell culture were collected after 24 h of treatment with TLR agonists, centrifuged to remove potential debris, and stored at − 20 °C. Undiluted supernatants were evaluated with enzyme-linked immunosorbent assay (ELISA) for cytokines TNFα, IL-6, und IL-1β. Porcine-specific ELISA Quantikine kits (R&D Systems, Minneapolis, MN, USA) were used for all cytokines. All ELISA kits were used according to the manufacturer’s instructions. Each experiment was independently repeated 5–6 times.

### Statistics

Each experiment was independently repeated at least 3 times. Statistical significance was assessed with the Kruskal–Wallis test and Mann–Whitney *U* test, with the exception of TER measurements and comparison of basal and apical cytokine secretion, for which significance was evaluated with a Wilcoxon matched-pairs signed rank test (for repeated measurements). A *p* value of < 0.05 was considered significant. Calculations were done in Microsoft Excel and GraphPad Prism 7.0. Graphs depict mean and standard deviation.

## Results

### Effect of TLR agonists on cell viability

**Fig. 2:**
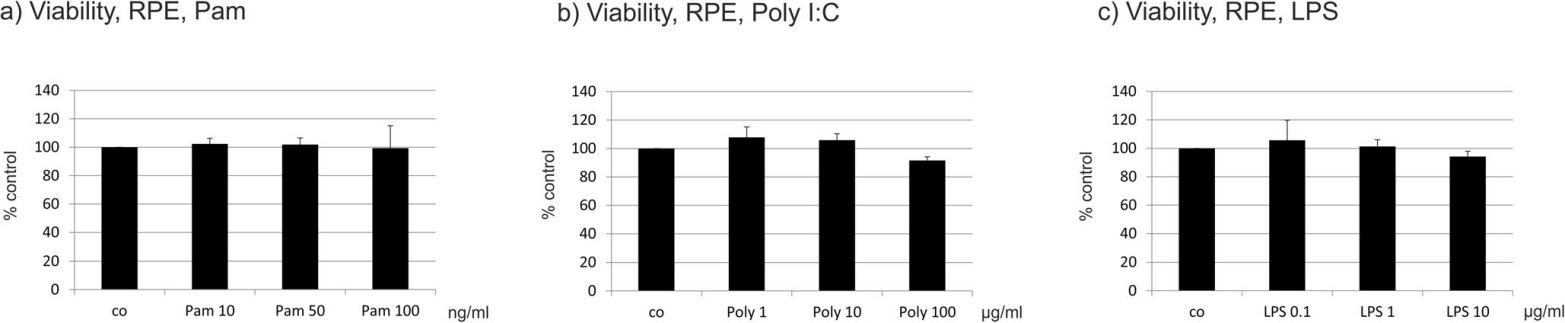
Viability of RPE cells after treatment with TLR agonists Pam (**a**), Poly I:C (**b**), or LPS (**c**) for 24 h. None of the applied agonists altered the viability of RPE cells (*n* = 3). Significance evaluated with the Kruskal–Wallis test

We have tested the effect of Pam, Poly I:C, and LPS on cell viability for the respective treatments. None of the tested substances displayed any effect on the viability of RPE cells (Fig. 2a–c).

### Cytokine secretion

#### Primary RPE

**Fig. 3:**
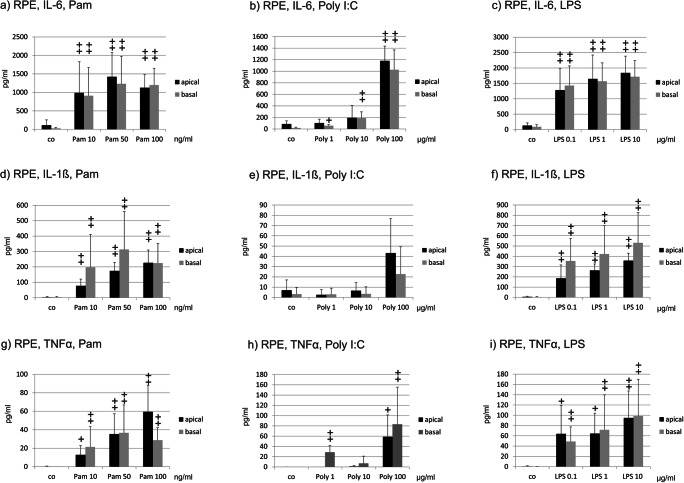
Apical and basal cytokine secretions of primary RPE cells after stimulation with TLR agonists. IL-6 was apically and basally induced by Pam (**a**) and LPS (**c**) at all concentrations tested. Poly I:C (**b**) induced basal secretion of IL-6 at all concentrations, while only 100 μg/ml induced apical secretion. IL-1ß was apically and basally induced by Pam (**d**) and LPS (**f**) at all concentrations tested, but not by Poly I:C (**e**). TNFα was apically and basally induced by Pam (**g**) and LPS (**i**) at all concentrations tested, while Poly I:C induced a basal secretion at 1 and 100 μg/ml and an apical secretion at 100 μg/ml (**h**). Differences between apical and basal secretions were not significant after any treatment tested (*n* = 5). Significance evaluated with the Kruskal–Wallis and Mann–Whitney *U* test, ^+^*p* < 0.05, ^++^*p* < 0.01, compared with control, and with the Wilcoxon matched-pairs signed rank test for comparison between apical and basal

Unstimulated RPE cells secreted only minor amounts of IL-6. Pam significantly induced IL-6 apically and basally at all concentrations tested (Fig. 3a). Poly I:C showed significantly increased secretion at the basal side at all concentrations tested, while at the apical side, only the highest concentration tested (100 μg/ml) induces a significant response (Fig. 3b). Of note, at 100 μg/ml Poly I:C IL-6 was much stronger induced on the basal side than at any other concentrations tested. LPS significantly induced IL-6 apically and basally at all concentrations tested (Fig. 3c). Only minute amounts of IL-1ß could be found in unstimulated primary RPE cells. Pam induced a significant increased secretion of IL-1ß apically and basally at all concentrations tested (Fig. 3d). Poly I:C did not display any significant influence on IL-1ß secretion (Fig. 3e). LPS induced a significant increase apically as well as basally at all concentrations tested (Fig. 3f). TNFα was significantly induced by Pam both apically and basally at all tested concentrations (Fig. 3g), while Poly I:C induced a basal secretion at 1 and 100 μg/ml and an apical secretion at 100 μg/ml (Fig. 3h). LPS significantly induced TNFα basally and apically at all tested concentrations (Fig. 3i).

#### RPE/choroid organ culture

**Fig. 4.**
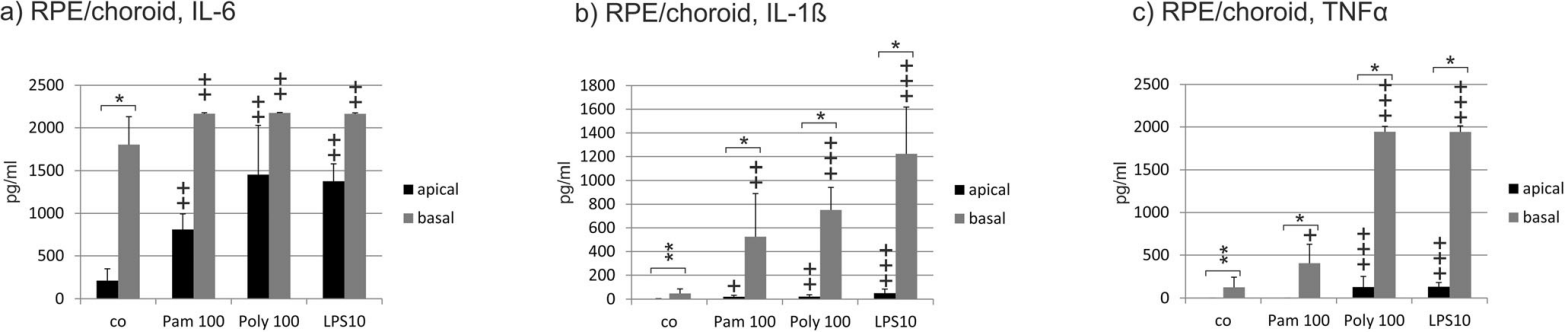
Apical and basal cytokine secretions of RPE/choroid organ cultures after stimulation with TLR agonists. **a** IL-6 was constitutively secreted on the basal but not apical side in RPE/choroid organ cultures. Pam, Poly, and LPS induced the secretion on the apical and basal sides. Between the apical and basal sides, no significant differences could be found. **b** In untreated RPE/choroid organ culture, only minute amounts of IL-1ß could be found on the basal side. Pam, Poly I:C, and LPS significantly induced IL-1ß on both sides, however, to a much stronger degree on the basal side. The difference between the apical and basal sides are statistically significant. **c** Only small amounts of TNFα could be found on the basal side. Poly I:C and LPS significantly induced TNFα on both sides; however, the induction was much stronger on the basal side. Pam induced TNFα on the basal side only. The differences between basal and apical secretions are statistically significant (*n* = 6). Significance evaluated with the Kruskal–Wallis and Mann–Whitney *U* tests, ^+^*p* < 0.05, ^++^*p* < 0.01, compared with control, and with the Wilcoxon matched-pairs signed rank test for comparison between apical and basal, *p < 0.05, **p < 0.01. Pam 100, Pam 100 ng/ml; Poly 100, Poly I:C 100 μg/ml; LPS 10, LPS 10 μg/ml

In untreated RPE/choroid organ culture, high amounts of IL-6 were secreted on the basal but not on the apical side. Pam, Poly I:C, and LPS significantly induced IL-6 on both sides. A statistically significant difference between the apical and basal sides was found for the untreated control only (Fig. 4a). Conversely, only minor amounts of IL-1ß can be found on the basal side of untreated RPE/choroid organ cultures. While all three agonists significantly induced IL-1ß on both sides, the induction on the basal side was much stronger. The difference in secretion between the apical and basal sides was statistically significant for all treatments as well as for the untreated control (Fig. 4b). Untreated RPE/choroid organ cultures secreted only small amounts of TNFα on the basal side. Pam induced TNFα significantly on the basal side only. Stimulation with Poly I:C and LPS, respectively, showed a significant induction on both sides, but the induction is much stronger on the basal side. The difference between the apical and basal side was statistically significant for all treatments as well as for the untreated control (Fig. 4c).

### Transepithelial resistance

**Fig. 5:**
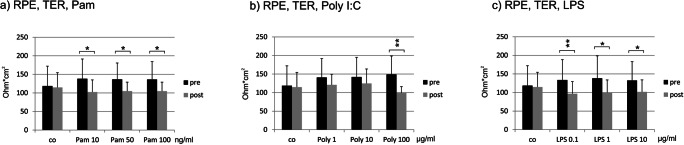
Transepithelial electric resistance (TER) of primary RPE cells after treatment with **a** Pam, **b** Poly I:C, and **c** LPS. Pam and LPS reduced the TER at all concentrations tested, while Poly I:C showed a significant reduction after treatment with 100 μg/ml (*n* = 5). Significance evaluated with the Wilcoxon matched-pairs signed rank test for comparison between pre- and post-treatment, *p < 0.05, **p < 0.01

Constituting the outer blood–retinal barrier is a major function of the RPE. Barrier function was investigated by TER measurements. Treatment with Pam for 24 h significantly reduced the TER at all concentrations tested (Fig. 5a). The effect of Poly I:C was less pronounced, with only 100 μg/ml reducing the TER significantly (Fig. 5b). Conversely, LPS significantly reduced TER at all tested concentrations (Fig. 5c).

### Occludin expression

Occludin is a tight junction protein important for barrier function. We investigated the expression of occludin in immunofluorescence and quantified the intensity and the distribution of the signal.

#### Primary RPE

**Fig. 6:**
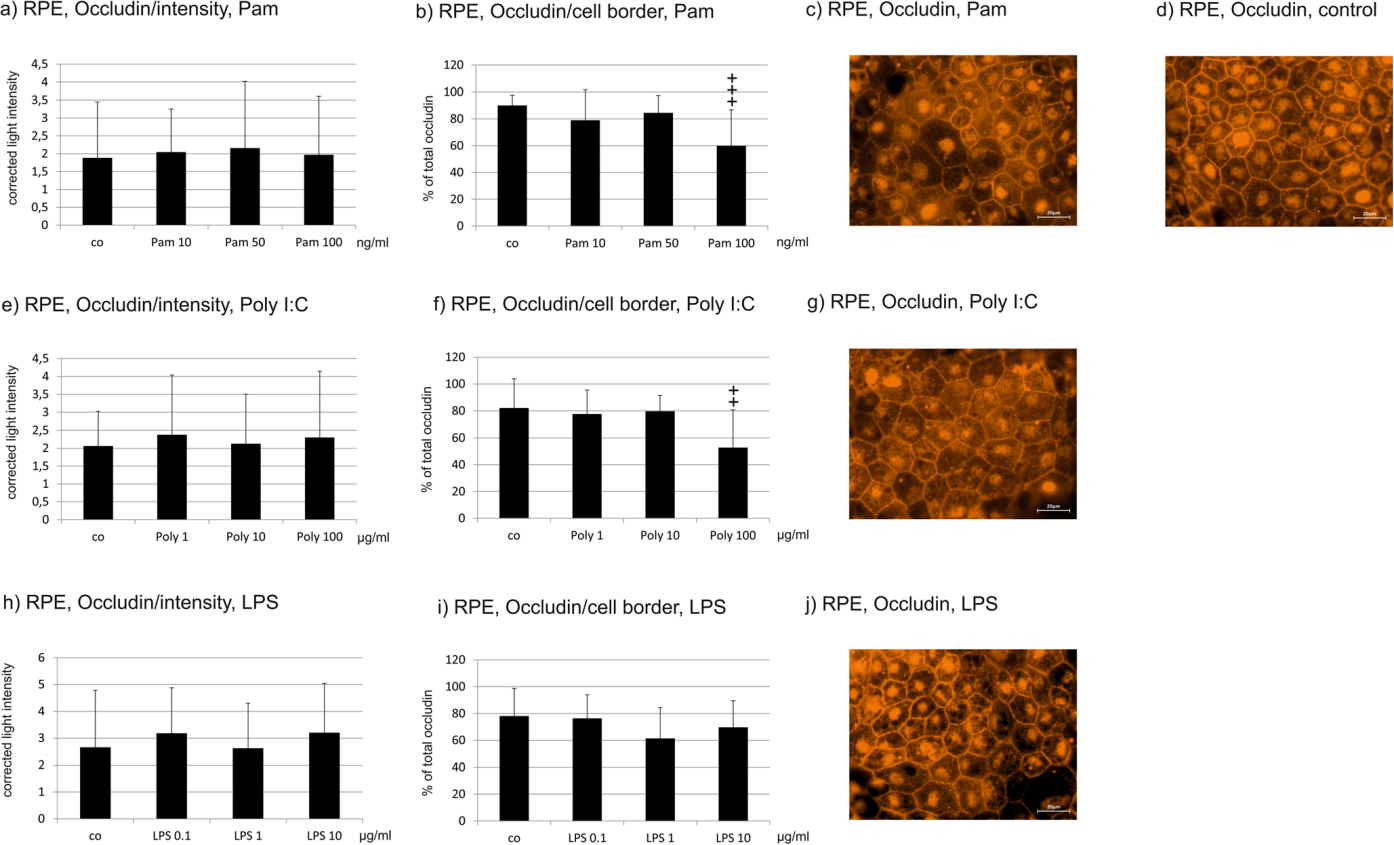
Occludin expression in primary porcine RPE cells after treatment with TLR agonists for 24 h. None of the agonists influenced the intensity of the occludin signal (**a**, **e**, **h**). Pam (**b**) significantly influenced the localization of occludin at a concentration of 100 ng/ml. Poly I:C (**f**) significantly reduced the amount of occludin found at the cell border at a concentration of 100 μg/ml. LPS (**i**) did not significantly alter the distribution. Representative examples are shown in **c** Pam 100 ng/ml, **g** Poly I:C 100 μg/ml, **j** LPS 10 μg/ml, and **d** control. Scale bar indicates 20 μm (*n* = 15). Significance evaluated with the Kruskal–Wallis and Mann–Whitney *U* tests, ^+^*p* < 0.05, ^++^*p* < 0.01, ^+++^*p* < 0.001, compared with control

None of the TLR agonists changed the intensity of occludin expression in the cells (Fig. 6a, e, h). Immunofluorescent occludin pictures showed a reduction of the amount of occludin at the cell borders (Fig. 6c, d, g, j), which was highly significant in semi-quantitative analysis for Pam at a concentration of 100 ng/ml (Fig. 6b) and Poly I:C at a concentration of 100 μg/ml (Fig. 6f). LPS showed no significant influence on occludin distribution in primary RPE (Fig. 6i).

#### RPE/choroid organ culture

**Fig. 7:**
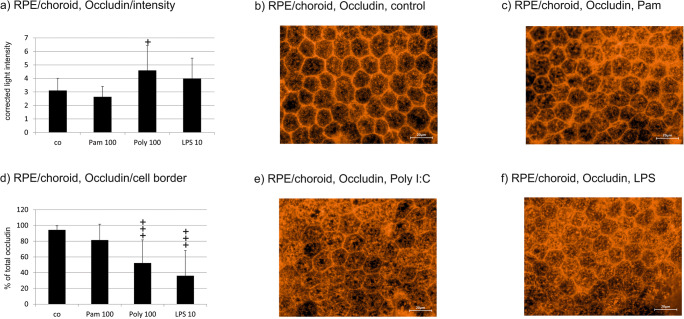
Occludin expression in RPE/choroid organ culture after treatment with TLR agonists for 24 h. Poly I:C but none of the other agonists significantly influenced the intensity of the occludin signal (**a**). Immunofluorescent occludin pictures show a redistribution of occludin into the cell body (**b** control, **c** Pam 100 ng/ml, **e** Poly I:C 100 μg/ml, **f** LPS 10 μg/ml), which is highly significant in semi-quantitative analysis for Poly I:C and LPS (**d**). Representative examples are given in **b** control, **c** Pam, **e** Poly I:C, and **f** LPS. Scale bar indicates 20 μm (*n* = 15), Significance evaluated with the Kruskal–Wallis and Mann–Whitney *U* tests, ^+^*p* < 0.05, ^+++^*p* < 0.001, compared with control. Pam 100, Pam 100 ng/ml; Poly 100, Poly I:C 100 μg/ml; LPS 10, LPS 10 μg/ml

The intensity of the occludin signal was significantly elevated by Poly I:C, while the other agonists showed no effect (Fig. 7a). Immunofluorescent occludin pictures showed a redistribution of occludin to the cell body (Fig. 7b, c, e, f), which was significant in semi-quantitative analysis for Poly I:C and LPS (Fig. 7d).

### Influence of supernatants on survival on SHSY-5Y cells

**Fig. 8:**
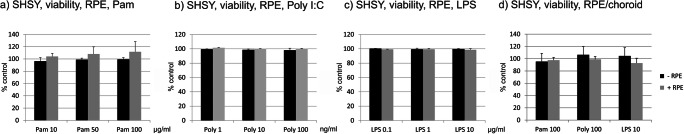
Viability of SHSY-5Y cells after treatment with TLR agonists (black bars) or supernatant of RPE (**a**–**c**) or RPE/choroid organ cultures (**d**), treated with TLR agonists (gray bars). Viability of SHSY-5Y was not affected by any treatment assessed in this assay (*n* = 3). Significance evaluated with the Kruskal–Wallis and Mann–Whitney *U* tests

We investigated whether the treatment of RPE cells or RPE/choroid organ culture with the TLR agonists exerted an effect on the survival on SHSY-5Y cells. Neither the direct treatment with the agonists nor the supernatant of treated RPE cells nor the supernatant of RPE/choroid organ cultures influenced the survival of the SHSY-5Y cells (Fig. 8).

## Discussion

In our study, we investigated the effect of the activation of pattern recognition receptors TLR2, 3, and 4 of the RPE on its barrier function and polarized cytokine secretion. We have used two different model systems: primary RPE cells, used without passaging, and RPE/choroid explants. Both were prepared from the pig. The pig is an excellent model for the human eye, which is closer to the human situation than rodents in general and specifically concerning the retina. The porcine eye is anatomically very similar to the human eye, and, contrary to mice, pigs are diurnal animals with a cone dense visual streak [42–44]. As the cells and organ cultures were prepared from genetically heterogenic pigs that were raised in a non-sterile farm environment, the standard deviation may be higher than what is usually expected when cell lines or mouse strains are used. However, the conditions used in this study reflect the real-life condition more closely [45].

In our study, the barrier properties were under investigation; therefore, we cultivated the primary RPE cells on transwell plates until a stable TER was established. The mean stable TER was 140.8 + 55.4 Ω*cm^2^, which is in the range of TER published for adult human RPE cells [46]. Of note, fetal RPE cells can achieve much higher TER [47]; however, these do not model the situation of an adult RPE layer subjected to inflammatory insults in the context of age-related macular degeneration [32]. In addition, occludin staining showed distinct cell borders in untreated control, indicating a functioning barrier. Furthermore, we have used primary cells without passaging. These cells are showing a higher degree of pigmentation and RPE morphology compared with passaged cells [32]. Moreover, the cytokine secretion profile changes with passaging; therefore, the use of cells without passaging is advised when determining cytokine secretion [32, 48]. In contrast to RPE cell cultures, RPE/choroid organ cultures still contain Bruch’s membrane and the choroid. Furthermore, the cultivation of RPE/choroid explants does not include detachment of the RPE monolayer; therefore, the barrier properties are not disturbed during preparation. Culturing RPE cells in transwell plates and RPE/choroid organ cultures in Ussing chambers enables a distinction between the apical and basal secretions [8, 9].

Unstimulated primary RPE cells secreted only minute amounts of IL-6, IL-1ß, or TNFα. In contrast, RPE/choroid organ cultures displayed a profound constitutive secretion of IL-6 to the basal but not to the apical side. For primary RPE cells, Pam as well as LPS induced the three investigated cytokines, IL-6, IL-1ß, and TNFα at all concentrations tested, with IL-6 being the most strongly induced (> 1000 pg/ml), IL-1ß to a lesser extent (between 100 and 200 pg/ml), and TNFα to a lower extent (< 100 pg/ml). Poly I:C reached similar amounts of IL-6 and TNFα only in the highest concentration studied (100 μg/ml), while IL-1ß was not significantly induced at all. A significant difference between apical and basal secretions could not be seen in primary RPE cells. For the RPE/choroid organ culture experiments, we used the highest concentrations of each TLR agonist. Here, all agonists induced IL-6 on both sides, and IL-1ß and TNFα were strongly induced on the basal side and only to a minor extent on the apical side. The differences between the apical and basal secretions were significant for all agonists tested. Contrary to our findings, in a study using commercially available primary human RPE cells and the ARPE-19 cell line, a predominantly apical secretion of IL-1ß has been shown [49]. However, in that study, the inflammatory response was induced by inflammasome activation, not TLR agonists. While not stated directly, the stimulation was likely to have been conducted on the apical, not the basal, side, as it was done in our study. An inflammasome activation on the apical side of the RPE is likely to induce a different polarization of cytokine secretion than a basal stimulation of TLR. This is in accordance with a study investigating the secretion of IL-6 and IL-8 after apical or basal stimulation with IL-1ß, showing that a basal stimulation would induce a stronger secretion to the basal side [50].

RPE cells respond to pro-inflammatory activation with cytokine secretion. The exact distribution and degree of the secretion depend on the exact insult [11, 23, 24, 51]. It is intriguing that the RPE/choroid reacted primarily at the basal side when (basally) stimulated. Furthermore, the response was different for the different cytokines tested—IL-6 is also induced on the apical side, while IL-1ß and TNFα are clearly mainly induced on the basal side. The basal response of the RPE/choroid either may be attributed to the side of stimulation—a basal stimulation may induce a stronger basal response [50]—or may be an attempt of the RPE to regulate the inflammatory response in order to protect itself. Both TNFα and IL-1ß are considered to be neuro-toxic, especially in damaged tissue [52]. IL-6, on the other hand, can have neuro-protective and neuro-regenerative effects in the retina [53–55]. This is in line with our previous studies regarding the efforts of the RPE to regulate the inflammatory response in communication with microglia or macrophages, where the RPE induced a differentiated pro-inflammatory response in microglia [13] or a dampening effect on macrophages [45]. We believe that the RPE is strongly involved in the regulation of the inflammatory response in the retina, balancing an effective response to the danger signal with the need to protect the retinal tissue from damage because of inflammation.

As pointed out above, we only find this polarized response in RPE/choroid organ cultures, not in primary RPE cells. This could be explained by a lack of barrier function and an inability of the cultured RPE cells to establish a polarized secretion. However, as pointed out above, our TER is adequate for adult RPE cells, and our occludin staining suggests functioning tight junctions [46]. Moreover, a polarized secretion of cytokines such as IL-6 has been shown for cells with a far lower TER (20 Ω*cm^2^) [50]. The most likely explanation for the difference in polarized secretion between the primary cells and the RPE/choroid organ culture is the interaction of the RPE with the choroid in the organ culture, which may enable the cells to react in a manner resembling the in vivo situation [38]. However, it has to be considered also that the choroid contains cell types that may react to TLR stimulation. Choroidal melanocytes and choroidal endothelial cells also express TLRs [56, 57], and macrophages may be present in the choroidal tissue [58]. The influence of the choroid on the inflammatory responses of the RPE and the involvement of choroidal cells regarding the inflammatory response are exciting topics that should be investigated more closely in further studies. Our data shows the importance of the interaction of the three-dimensional tissue especially when it comes to complex orchestration of tissue responses.

In accordance with the proposed attempted protection of the retina by the RPE even under inflammatory condition, we did not find any cytotoxic effect of RPE supernatants on SHSY-5Y cells. It has to be pointed out that both IL-1ß and TNFα can exceed different effects depending on the duration of the stimulus, with long-term stimulation associated with neurotoxicity, and we tested only for 24 h [59, 60]. Therefore, long-term effects may differ.

Our results show that pro-inflammatory stimulation with TLR agonists impedes barrier function of RPE cells and may have an impact on occludin distribution. Our data is in accordance with previously published data describing a reduction of the TER of ARPE-19 cells treated with LPS [61]. Still, the aspect of barrier function after TLR activation has received little attention so far. Of note, TNFα has also been shown to reduce the TER of RPE cells [62, 63]. TNFα is induced by our agonists, indicating that TNFα may be involved in the TER alterations. Of note, however, TNFα was reported to be effective only when applied to the apical side and to act independently from occludin [62] or to reduce occludin expression [63]. Therefore, an involvement of TNFα in TER alterations in our setting is possible, but other mechanisms may also be involved. Further research is needed to elucidate the exact mediators and pathways influencing the barrier functions after TLR activation. Impeding the barrier function of the RPE can be of consequence for the retina. The outer blood–retinal barrier regulates the exchange of metabolites and waste products between the choroid and the photoreceptors [64]. Furthermore, the outer blood–retinal barrier is important for the immune privilege of the retina and protects the retina from unwanted intrusion from the systemic circulation [65]. A disrupted barrier may interrupt the regulated exchange between the choroid and the retina. Furthermore, it may facilitate the intrusion of blood-borne leukocytes into the retina, boosting the inflammatory responses and retinal degeneration [66]. AMD is associated with the disruption of the blood–retinal barrier, and the alteration of the barrier by pro-inflammatory activation may be an important factor in AMD disease progression [67]. More research is warranted to further elucidate the influence of TLR activation on the barrier function. In addition to looking at occludin, the influence of TLR activation on other tight junction proteins, such as ZO-1 or claudin 19, should be further investigated, looking at localization as well as protein expression. In addition, the consequences concerning transport of molecules and therapeutics (such as anti-VEGF medication when considering AMD) across the barrier should be elucidated further. Furthermore, the mechanisms of barrier disruption need to be further investigated, e.g., determining the contribution of specific cytokines such as TNFα of IL-6 by specifically blocking them.

In our study, we have applied the stimulus on the basal side of the RPE to simulate a systemic inflammation. Our data support the hypothesis that systemic inflammation may contribute to the development of AMD [25, 26], as our basal (corresponding to the side of the choroid) pro-inflammatory stimuli induce pro-inflammatory cytokine release in the RPE and a disruption of the RPE barrier function. These results are also supported by a recent in vivo study that showed that a systemic virus infection can alter the expression of chemokines and complement factors in the RPE/choroid [68].

Taken together, our study shows that TLR activation of RPE cells induced a polarized cytokine secretion and reduced the barrier function of the RPE cells, while displaying no toxicity. Polarized cytokine secretion discloses another layer of the involvement and regulation of the RPE in retinal inflammation. Inflammation-induced loss of barrier function might constitute a mechanism of pathogenesis in AMD. Further research is needed to elucidate the effect of inflammation of the outer blood–brain barrier.

## Data Availability

The data is available upon request.
